# Anthropomorphic Tendon-Based Hands Controlled by Agonist–Antagonist Corticospinal Neural Network

**DOI:** 10.3390/s24092924

**Published:** 2024-05-03

**Authors:** Francisco García-Córdova, Antonio Guerrero-González, Fernando Hidalgo-Castelo

**Affiliations:** Department of Automation, Electrical Engineering, and Electronic Technology, Polytechnic University of Cartagena, 30203 Cartagena, Spain; fgc_master@upct.es (F.G.-C.); fernando.hidalgo2@edu.upct.es (F.H.-C.)

**Keywords:** corticospinal neural network, neurobiological control, anthropomorphic robotic systems, agonist–antagonist control, dynamic neural network, multi-finger robotic hands

## Abstract

This article presents a study on the neurobiological control of voluntary movements for anthropomorphic robotic systems. A corticospinal neural network model has been developed to control joint trajectories in multi-fingered robotic hands. The proposed neural network simulates cortical and spinal areas, as well as the connectivity between them, during the execution of voluntary movements similar to those performed by humans or monkeys. Furthermore, this neural connection allows for the interpretation of functional roles in the motor areas of the brain. The proposed neural control system is tested on the fingers of a robotic hand, which is driven by agonist–antagonist tendons and actuators designed to accurately emulate complex muscular functionality. The experimental results show that the corticospinal controller produces key properties of biological movement control, such as bell-shaped asymmetric velocity profiles and the ability to compensate for disturbances. Movements are dynamically compensated for through sensory feedback. Based on the experimental results, it is concluded that the proposed biologically inspired adaptive neural control system is robust, reliable, and adaptable to robotic platforms with diverse biomechanics and degrees of freedom. The corticospinal network successfully integrates biological concepts with engineering control theory for the generation of functional movement. This research significantly contributes to improving our understanding of neuromotor control in both animals and humans, thus paving the way towards a new frontier in the field of neurobiological control of anthropomorphic robotic systems.

## 1. Introduction

The dexterity and versatility of the human hand have fascinated scientists and engineers for decades. As one of the most important tools of the human body, the hand is capable of performing a wide range of movements and manipulating objects with remarkable precision and agility [1]. This dexterity is due to its complex anatomical structure, which includes multiple joints, tendons, and muscles, as well as the sophisticated neural control mechanisms that coordinate these components [2].

In the field of robotics, the human hand has served as an important source of inspiration for the development of robotic limbs and manipulation devices [3]. Researchers have sought to replicate the capabilities of the human hand in anthropomorphic robotic systems with the goal of achieving similar levels of dexterity and adaptability [4]. However, designing and controlling robotic hands that can match the performance of the human hand remains a significant challenge due to the mechanical and computational complexity involved [5].

One of the main difficulties in the development of anthropomorphic robotic hands is the creation of mechanical systems that can replicate the kinematics and dynamics of the human hand [6]. The human hand has more than 20 degrees of freedom (DoF), allowing it to perform a wide variety of movements and postures [7]. Additionally, the hand is equipped with a network of tendons and muscles that provide strength and allow for precise and coordinated movements [8]. Replicating this complexity in a robotic system requires careful mechanical design and the integration of advanced actuators and sensors [9].

Another major challenge is the development of control algorithms that can effectively coordinate the multiple degrees of freedom of a robotic hand to perform manipulation tasks [10]. The movements of the human hand are controlled by a complex network of neural circuits in the brain and spinal cord, which continuously integrate sensory information and generate appropriate motor commands [11]. These neural circuits enable the human hand to adapt to different grasp conditions, compensate for disturbances, and learn new skills through practice [12]. Replicating these capabilities in a robotic control system requires sophisticated algorithms that can handle the nonlinear dynamics of the hand and adapt to changing environments [13].

In recent decades, significant advances have been made in the development of anthropomorphic robotic hands. Researchers have proposed various mechanical designs that attempt to replicate the structure and function of the human hand, using approaches such as 3D printing, flexible actuators, and soft polymer materials [14]. These designs have demonstrated impressive dexterity and adaptability in manipulation tasks, but still fall short of matching the complexity and performance of the human hand [15].

In addition to advances in mechanical design, there has also been significant progress in the development of control algorithms for anthropomorphic robotic hands. Traditional approaches, such as impedance control and force control, have been widely used to control robotic hands during manipulation tasks [16]. More recently, advances in machine learning and neural networks have led to the development of more flexible and adaptable control algorithms that can learn from experience and adapt to new tasks [17].

Despite these advances, there are still many challenges to overcome in order to create robotic hands that can match the capabilities of the human hand. One of the main challenges is the integration of tactile and proprioceptive sensors that can provide real-time feedback on contact forces and hand position [18]. This sensory feedback is crucial for precise control of grip force and manipulation of delicate objects [19]. Another challenge is the development of control algorithms that can handle the complex dynamics of real-world manipulation tasks, such as manipulation of deformable objects, manipulation in the presence of uncertainty, and force control in cluttered environments [20].

To address these challenges, many researchers have turned to neuroscience and physiology for insights into how the human nervous system controls the hand [21]. Studies of the motor cortex, cerebellum, and basal ganglia have shed light on the neural mechanisms involved in controlling joint movements [22]. Additionally, studies of the spinal cord and reflex circuits have provided insights into how motor commands are generated and coordinated at a local level [23]. These insights have inspired the development of neuromorphic and biologically inspired control architectures for robotic structures that seek to replicate the function of biological neural circuits [24].

A particularly interesting approach to controlling robotic hands is the use of bioinspired control algorithms that are based on computational models of biological neural circuits [25]. These algorithms seek to replicate the principles of information processing and motor command generation found in the nervous system, with the goal of achieving more natural and adaptable control of robotic hands [26].

Based on the equilibrium point theory, several researchers have proposed control architectures that use muscle and tendon models to generate movements [27]. These models typically represent muscles as nonlinear elastic elements that generate force as a function of their length and contraction velocity [28]. By adjusting the resting lengths of these muscle models, the controller can specify different joint postures and movements [29].

Another important concept from neuroscience that has inspired bioinspired control algorithms is the notion of muscle synergies [30]. Muscle synergies refer to spatiotemporal patterns of muscle activation that occur in a coordinated manner to produce movements [31]. The central nervous system is thought to simplify the control problem by using a limited set of muscle synergies that can be combined in various ways to generate a wide range of movements [32].

Several studies have demonstrated the presence of muscle synergies in the human hand during various grasping and manipulation tasks [33]. These findings have inspired the development of robotic controllers that utilize muscle synergies to generate hand movements [34]. In these approaches, synergies are typically implemented as a set of predefined muscle activation commands that can be linearly combined to produce different hand postures and movements [35].

In this context, the present study aims to contribute to the advancement of the field of anthropomorphic robotic hands through the development and experimental validation of a novel bioinspired control architecture for the control of a tendon-driven multi-fingered robotic hand. The proposed control architecture is based on computational models of the neural circuits in the motor cortex and spinal cord, and seeks to replicate the capabilities of dexterity, adaptability, and robustness of the human hand.

## 2. Materials and Methods

### 2.1. Anthropomorphic Robot Hand

**Figure 1:**
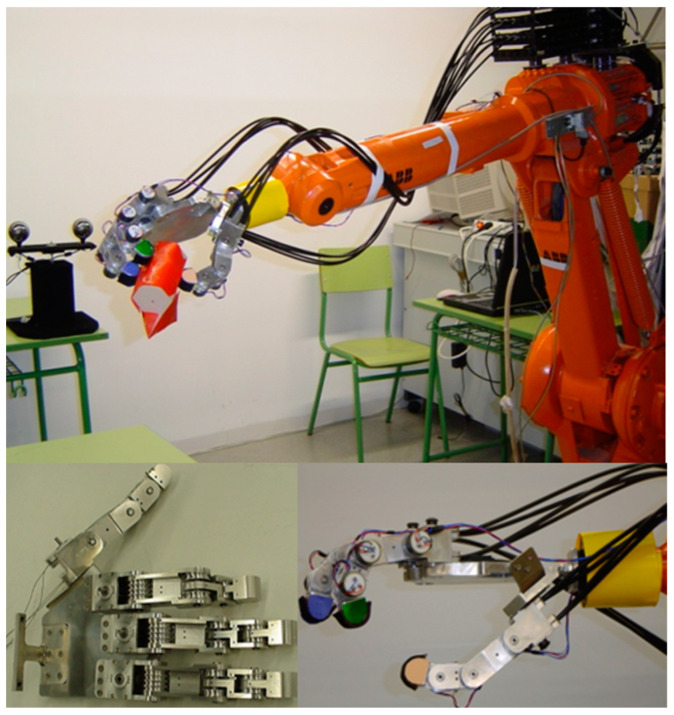
The design of the anthropomorphic robotic hand, “Cervantes’s hand”.

The anthropomorphic robotic hand, named the “Cervantes Hand” in honor of the Spanish writer Miguel de Cervantes Saavedra, was designed based on biomechanical modeling of the human hand. The hand has three fingers and an opposing thumb, all mounted on a rigid palm made of lightweight and durable aluminum. The dimensions of the hand are 269 (L) × 109 (W) × 26 (H) mm. Figure 1 shows the design of the anthropomorphic robotic hand and Table 1 details its main characteristics.

**Figure 2:**
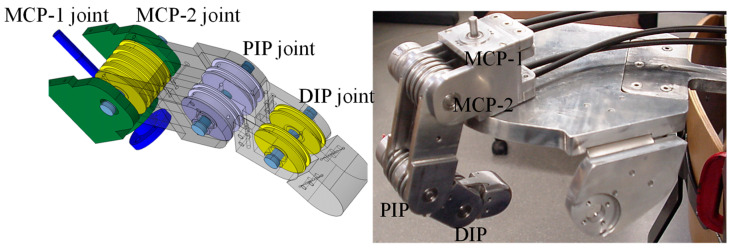
The design of the multi-jointed robotic finger.

The design of the robotic fingers, which is identical for both the fingers and the thumb, incorporates four joints: the first metacarpophalangeal (MCP_1_), the second metacarpophalangeal (MCP_2_), the proximal interphalangeal (PIP), and the distal interphalangeal (DIP). The dimensions of the fingers are 158 (L) × 26 (W) × 25 (H) mm, as shown in Figure 2. In the robotic finger, Hall-effect position sensors are integrated into each joint to accurately measure the position, velocity, and direction of rotation of the joint. Additionally, tactile sensors, specifically force sensing resistors (FSR), are installed on the fingertips and palm.

**Figure 3:**
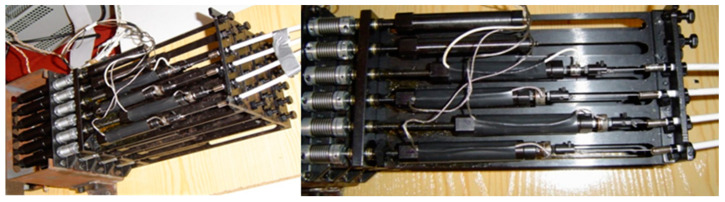
Artificial muscles of the anthropomorphic robot finger.

The PIP and DIP joints are designed for flexion and extension movements, while the MCP joint consists of two separate joints, MCP_1_ and MCP_2_, which allow for abduction–adduction and flexion–extension movements, respectively. The finger design incorporates muscle-like actuators, represented by direct current (DC) motors which are combined with lead screws to produce linear movements. Each joint is driven by two braided stainless-steel cables coated with nylon, which function as tendons and are powered by DC motors equipped with lead screws, as shown in Figure 3. The DIP joint is mechanically coupled to the PIP joint through a system of pulleys and tendons, creating a rotational relationship defined as *rc* = *r* 13 *r* 15. Table 2 details the technical specifications of the robotic fingers, focusing on joint mobility.

**Figure 4:**
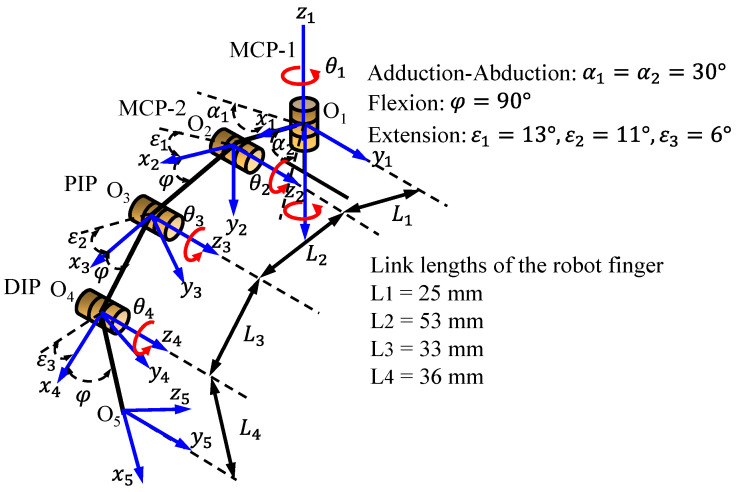
Representation of the assignment of reference coordinate systems of the kinematic chain of the robot finger.

The positions and orientations of the fingertip are determined by the joint angles, link lengths, and the location of the coordinate frames at the joints. These parameters are defined using the Denavit–Hartenberg (DH) convention, as illustrated in Figure 4 and detailed in Table 3. The DH parameters provide a systematic approach to describe the kinematic relationship between the joints and links of the robotic finger.

- θ_i_: rotation angle around the z_i−1_ axis to align x_i−1_ with x_i_.
- d_i_: distance along the z_i−1_ axis from the origin of the coordinate system (i−1) to the intersection of the z_i−1_ axis with x_i_.
- a_i_: distance along the xi axis from the intersection of the z_i−1_ with x_i_ to the origin of the i.
- α_i_: rotation angle around the xi axis to align z_i−1_ with z_i_.

The rotation angles (θ_i_) are the joint variables and depend on the specific configuration of the finger at a given time. The displacement parameters (d_i_) and torsion angles (α_i_) are set to zero, except for the MCP_1_ joint, where α1 is set to π/2 to represent the perpendicular orientation between the rotation axes of MCP_1_ y MCP_2_.

Using the DH parameters, we can develop the direct and inverse kinematic equations relating the joint angles to the position and orientation of the robotic finger end-effector.

**Figure 5:**
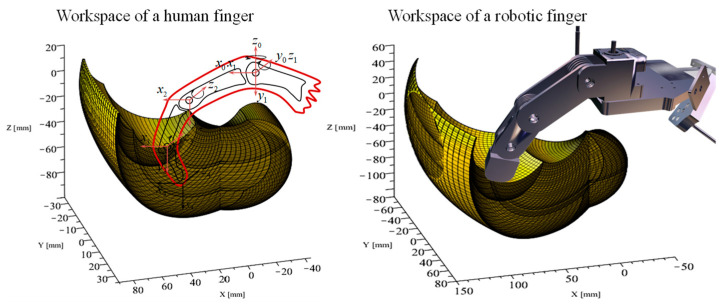
Workspace distribution for different spatial configurations of a robotic finger and a human finger model.

The behavior of the 4 degrees of freedom (DoF) anthropomorphic robotic finger closely resembles that of a human finger model in the workspace, as shown in Figure 5.

### 2.2. Actuation System of the Anthropomorphic Robotic Hand

**Figure 6:**
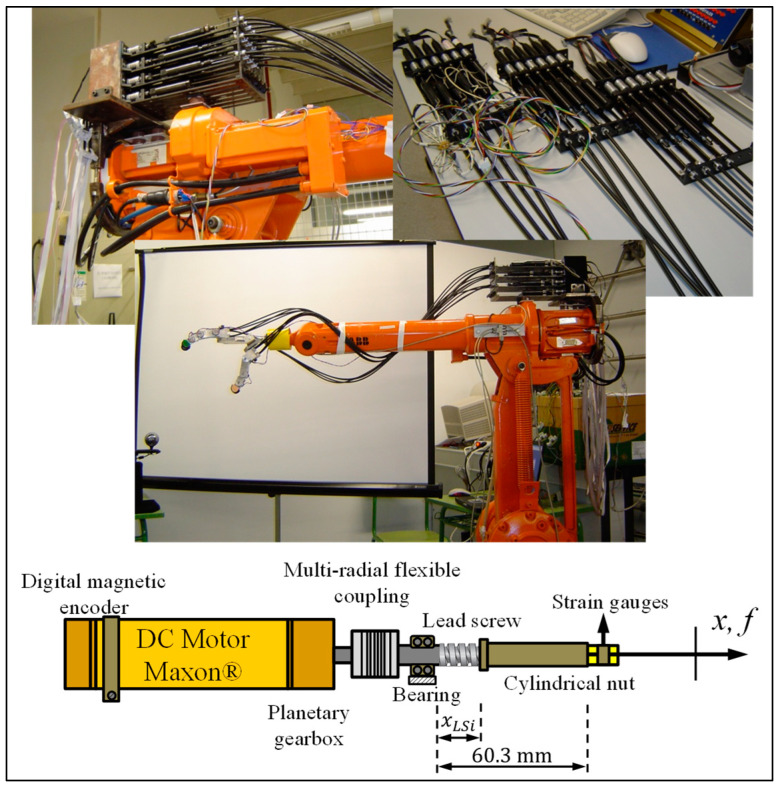
Robotic actuation system with fingers. The muscle-like artificial actuator has a DC motor with an elastic coupling followed by a lead screw to drive a tendon.

In each robotic finger, the joints are actuated by tendons connected to artificial muscles, which are composed of an elastic DC actuator with a digital magnetic encoder, a planetary gearbox, a multi-radial flexible coupling, a bearing, a lead screw, and a cylindrical nut connected to a force sensor (strain gauge). This design, illustrated in Figure 6, aims to emulate the behavior of human or animal muscles using DC micromotors coupled with a lead screw. The actuation system achieves a high force-to-mass and power-to-mass ratio, which facilitates stable force control with low impedance and a wide dynamic range.

#### 2.2.1. Dynamics of Artificial Muscles

**Figure 7:**
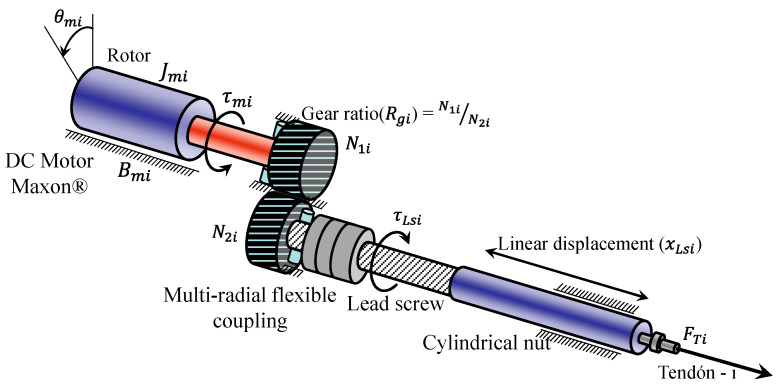
Dynamic parameters of muscle-like actuators.

The model of the muscle-like actuator is represented by a set of equations that describe the dynamics of the DC motor, gearbox, lead screw, and tendon force produced with respect to a load:

(1) $$\tau_{mi}=K_{i}i_{ai}$$

where $\tau_{mi}$ is

(2) $$\tau_{mi}=\left[J_{mi}+{\left[\frac{N_{1i}}{N_{2i}}\right]}^{2}J_{Lsi}\right]{\ddot{\theta }}_{mi}+B_{mi}{\dot{\theta }}_{mi}+\left[\frac{N_{1i}}{N_{2i}}\right]\left[\frac{a}{2\pi }\right]F_{Ti}$$

where $e_{bi}$ is

(3) $$V_{ai}=R_{ai}i_{ai}+L_{ai}\frac{di_{ai}(t)}{dt}+e_{bi}$$

(4) $$e_{bi}=K_{bi}{\dot{\theta }}_{mi}$$

where i=1,⋯,n+1 ó 2n (determined by the type of tendon routing in the robot finger), in our case i=1,⋯,6 representing the actuator number, $K_{i}$ is the motor torque constant, $i_{ai}$ is the armature current, $\tau_{mi}$ is the motor torque, $J_{mi}$ is the moment of inertia of the motor rotor, $\theta_{mi}$ is the angular velocity of the motor, $\omega_{mi}$ is the angular velocity of the motor, $B_{mi}$ is the coefficient of viscosity of the motor,$\;V_{ai}$ is the armature voltage of the motor, $R_{ai}$ is the armature resistance of the motor, $L_{ai}$ is the armature inductance of the motor, $e_{bi}$ is the power transmission ratio (EMF), $K_{bi}$ is the motor EMF constant, $R_{gi}=N_{1i}/N_{2i}$ is the gearbox ratio, $\tau_{Lsi}$ is the gearbox output torque introduced to the lead screw, $\omega_{Lsi}$ is the angular velocity of the lead screw, $J_{Lsi}$ is the moment of inertia of the lead screw, $R_{Lsi}=a_{i}/2\pi$ is the ratio of angular displacement to linear displacement, $a_{i}$ is the pitch of the lead screw, $x_{Lsi}$ is the linear displacement of the cylinder on the lead screw, $F_{Ti}$ is the tendon force produced by the motor, with respect to a load, and mi is the mass of the load. The parameter values used in experiments and simulations of the artificial muscle were $K_{i}=16.2$ mNm/A, $J_{mi}=1.23$ gcm^2^, $B_{mi}=3\times 10^{-5}$ Nms, $R_{ai}=11.2$ Ω (ohm), $L_{ai}=0.45$ mH, $K_{bi}=1.8\times 10^{-4}$ Vs/rad, $R_{gi}=24/1$, $J_{Lsi}=6\times 10^{-4}$ kgcm^2^, $a_{i}=1$ mm, ${\left(x_{Lsi}\right)}_{max}=60.3$ mm. The DC motors were Maxon DC Motor, RE 16, ∅16 mm, and precious metal brushes, 4.5 Watt. The dynamic parameters of the artificial muscles are plotted in a diagram shown in Figure 7.

#### 2.2.2. Joint and Actuator Space

**Figure 8:**
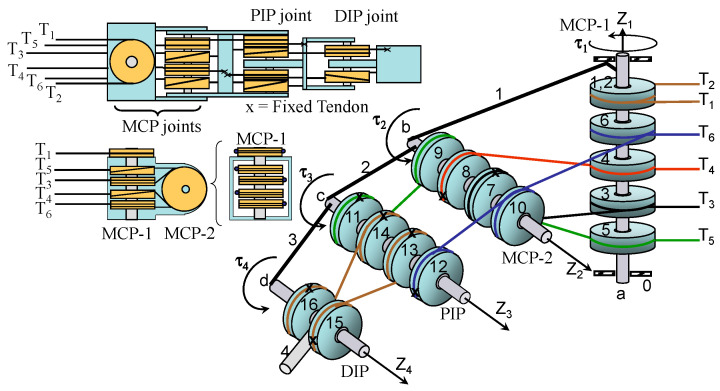
Functional representation of the anthropomorphic finger. Each joint is driven by two linear DC/tendon motors that emulate an agonist–antagonist muscle pair.

**Figure 9:**
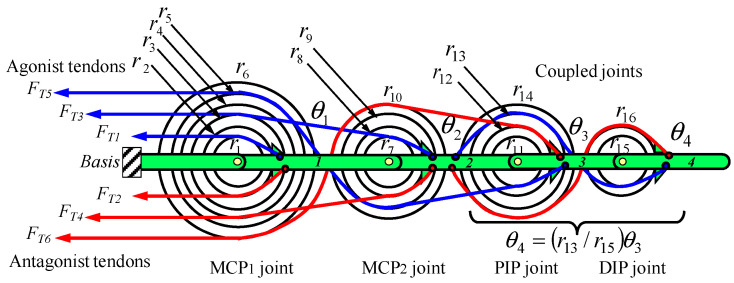
Four-link robotic finger and planar schematic representation of the tendon routing system. All pulleys rotate freely and the tendons are attached to the links of the robotic finger.

The functional representation of the anthropomorphic robotic finger is shown in Figure 8 and the planar schematic representation of the tendon routing system ($F_{Ti}$) is shown in Figure 9. The tendon configuration is a 2n-type coupled structure. The tendon-routed robotic finger uses six tendons and actuators, where each of the actuators pulls one tendon. Each independent joint is actuated by two tendon-actuators, which are arranged in opposite agonist–antagonist directions. As mentioned above, the DIP joint is coupled with the PIP joint at a ratio of $r_{c}=r_{13}/r_{15}=10/11$ mm.

The tensile force and displacement transformations between the joint space and tendon space are described by the following relationship:

(5) $$\tau \left(\theta ,\dot{\theta ,u}\right)=A^{T}F_{T},$$

and

(6) $$L=L_{0}-A\theta$$

where $A^{T}$ is

(7) $$A^{T}=\left[\begin{array}{c} r_{1}\\ 0\\ \begin{array}{c} 0\\ 0 \end{array} \end{array}\begin{array}{c} {\;-r}_{2}\\ \;0\\ \begin{array}{c} \;0\\ \;0 \end{array} \end{array}\begin{array}{c} {\;\;\;r}_{3}\\ {\;\;\;r}_{7}\\ \begin{array}{c} \;0\\ \;0 \end{array} \end{array}\begin{array}{c} -r_{4}\\ {-r}_{8}\\ \begin{array}{c} \;0\\ \;0 \end{array} \end{array}\begin{array}{c} \;\;r_{5}\\ {\;\;r}_{9}\\ \begin{array}{c} {\;\;\;r}_{11}\\ {\;\;\;r}_{11}r_{c1} \end{array} \end{array}\begin{array}{c} \;{-r}_{6}\\ {\;-r}_{10}\\ \begin{array}{c} -r_{12}\\ {\;\;-r}_{12}r_{c2} \end{array} \end{array}\right],\;\mathrm{where}\;r_{c1}=\left(\frac{r_{13}}{r_{15}}\right),\;r_{c2}=\left(\frac{r_{14}}{r_{16}}\right)$$

Muscle lengths ($L_{i}$) are obtained through Equation (6) and normalized from 0 to 1. In this specific case, the normalization of muscle lengths is obtained as follows:

(8) $$L_{1n}=\frac{L_{1}-L_{10}+r_{1}\theta_{1min}}{\left(\theta_{1min}-\theta_{1max}\right)r_{1}}$$

(9) $$L_{2n}=1-L_{1n}$$

(10) $$L_{3n}=\frac{L_{3}-L_{30}+r_{3}\theta_{1min}+r_{7}\theta_{2min}}{\left(\theta_{1min}-\theta_{1max}\right)r_{3}+\left(\theta_{2min}-\theta_{2max}\right)r_{7}}$$

(11) $$L_{4n}=1-L_{3n}$$

(12) $$L_{5n}=\frac{L_{5}-L_{50}+r_{5}\theta_{1min}+r_{9}\theta_{2min}++r_{11}\theta_{3min}}{\left(\theta_{1min}-\theta_{1max}\right)r_{5}+\left(\theta_{2min}-\theta_{2max}\right)r_{9}++\left(\theta_{3min}-\theta_{3max}\right)r_{11}}$$

(13) $$L_{6n}=1-L_{5n}$$

where the minimum and maximum angles [$\theta_{imin}$, $\theta_{imax}$] of the joints ($\theta_{i}$) are in the following ranges: MCP_1_$⟶\theta_{1}$ ∈ [−π/6, π/6], MCP_2_$⟶\theta_{2}$ ∈ [−π/2, 13π/180], PIP$⟶\theta_{3}$ ∈ [−π/2, 11π/180], and DIP$⟶\theta_{4}$ ∈ [−π/2, 6π/180].

As noted in the modeling of the system, we have assumed that the amount of stretch in each tendon due to stress variation is negligible. This assumption is due to the fact that

(a) The stiffness of the tendons is large ($\approx 43\times 10^{7}N/m^{2}$).
(b) The maximum tensile forces (of 219 N) generated by the DC micromotors and lead screw do not achieve a considerable expansion length in the tendons.
(c) The links have light weights that do not produce any elongation in the tendons. In addition, in the tendon drive system, passive prestressing was required in each tendon in order to ensure the tensions would remain under constant tension.

The parameters of the robotic finger using artificial linear actuators are $l_{1}=25$ mm, $l_{2}=53$ mm, $l_{3}=33$ mm, $l_{4}=36$ mm, $l_{ci}=l_{i}/2$, $m_{1}=0.0895$ kg, $m_{2}=0.0611\;$ kg, $m_{3}=0.048\;$ kg, $m_{4}=0.0415\;$ kg, $r_{13}=r_{14}=10$ mm and the remaining radii of $r_{i}=11$ mm. The passive resting lengths of the muscles are $L_{10}=L_{20}=$ 97.5 cm, $L_{30}=L_{40}=$ 100 cm and $L_{50}=L_{60}=$ 105.3 cm. 

The inverse transformation of Equation (5) can be written as:

(14) $$F_{T}={\left(A^{T}\right)}^{†}\tau +Hf_{0}$$

where ${\left(A^{T}\right)}^{†}={\left(A^{T}\right)}^{T}{\left[{\left(A^{T}\right)\left(A^{T}\right)}^{T}\right]}^{-1}$ is the pseudoinverse of $A^{T}$, H is a null space matrix m×(m−n) with its column vectors spanning the null space of $A^{T}$, and $f_{0}$ is an arbitrary (m−n)×1 vector representing a force bias (prestress) to avoid unnecessary negative forces in tendon traction. The first term on the right-hand side of the equation is known as the particular solution and the second term is the homogeneous solution. The homogeneous solution or the null space matrix satisfies

(15) $$A^{T}H=0$$

#### 2.2.3. End-Effector and Joint Space

The Jacobian matrix of the robotic finger maps the joint velocity vector $\dot{\theta }$ to the generalized velocity vector x ˙ of the end-effector:

(16) $$\dot{x}=J\left(\theta \right)\dot{\theta }.$$

The joint torques are related to an output force on the end-effector by the following equation:

(17) $$\tau =J{\left(\theta \right)}^{T}f$$

where f is an n×1 output force vector at the end effector:

(18) $$f={\left(f_{x},\;f_{y},\;f_{z},\;n_{x},n_{y},n_{z}\right)}^{T}$$

where $\left(f_{x},\;f_{y},\;f_{z}\right)$ are components of forces and $\left(n_{x},n_{y},n_{z}\right)$ are components of pairs at the fingertip.

#### 2.2.4. End-Effector and Actuator Spacing

Substituting Equation (17) into (14), we obtain

(19) $$F_{T}={\left(A^{T}\right)}^{†}\tau +Hf_{0}$$

The particular solution in this equation is the minimum norm solution, which could contain infeasible negative forces. However, homogeneous solutions can be controlled by adjusting ${\;f}_{0}$ to compensate for these negative forces so that the tendon stresses are always positive. Both the particular solution space (the column space of the matrix product ${\left(A^{T}\right)}^{†}J{\left(\theta \right)}^{T}$ and the homogeneous solution space (the column space of the matrix H) have significant effects on the hamstring forces. We can conclude that the configuration of the transmission structure (matrix A) is as important as that of the bond structure (matrix J).

### 2.3. Corticospinal Neural Network

The proposed corticospinal neural network model is specifically designed to control joint movements in anthropomorphic robotic fingers. The model is based on the simulation of cortical and spinal areas, as well as the connectivity between them during the execution of voluntary movements. It seeks to replicate the neural mechanisms involved in voluntary motor control, capturing the complex interactions between sensory and motor areas of the cerebral cortex, modulation of proprioceptive feedback, and dynamic compensation necessary to generate precise and adaptive movements.

#### 2.3.1. Model Architecture

The model consists of two main cortical areas: area 4 (the primary motor cortex) and area 5 (the posterior parietal cortex). These areas are interconnected and work together to generate motor commands and process sensory feedback.

In area 5, difference vector (DV) neurons compare the desired target position (TPV) with the perceived current position (PPV) to calculate the necessary changes in muscle lengths. The PPV is estimated from an efference copy of the position command from area 4 and error feedback from muscle spindles.

In area 4, desired velocity vector (DVV) neurons receive the DV signal modulated by a GO signal from the basal ganglia, controlling movement initiation and speed. Output position vector (OPV) neurons integrate the velocity command to generate a position command. Output force + position vector (OFPV) neurons combine the position command with static (SFV) and dynamic (IFV) force compensation signals to adapt to external loads. 

#### 2.3.2. Model Neurons

- Desired velocity vector (DVV) neurons: In area 4, the DV signal modulated by the GO signal is integrated to generate a velocity command.
- Output position vector (OPV) neurons: In area 4, the velocity command is integrated to produce a position command.
- Output force + position vector (OFPV) neurons: Additionally, in area 4, the position command is combined with force compensation signals to allow them to adapt to external loads.
- Perceived position vector (PPV) neurons: Located in area 5, the current position of driven joints is estimated based on an efference copy of the position command and sensory feedback from muscle spindles.

#### 2.3.3. Motor Neurons and Sensory Afferents

- Alpha motor neurons (α-MN): Motor commands from OFPV neurons in area 4 and extrafusal muscle fibers are activated to generate force and movement.
- Static gamma motor neurons (γs-MN): Innervation by OPV neurons in area 4 allows the sensitivity of muscle spindles to be adjusted by controlling the stiffness of intrafusal fibers.
- Dynamic gamma motor neurons (γd-MN): Input is received from DVV neurons in area 4 and modulate spindle sensitivity to velocity changes by controlling the viscosity of intrafusal fibers.
- Ia afferents: Primary sensory fibers from muscle spindles that detect changes in muscle length and velocity. These provide proprioceptive feedback to the central nervous system.
- II afferents: Secondary sensory fibers from muscle spindles that primarily detect changes in muscle length.

#### 2.3.4. Model Signals and Variables

- L1n, L2n, L3n, L4n: Normalized muscle lengths in the model. L1n and L2n correspond to the antagonist muscles controlling the proximal interphalangeal (PIP) joint, while L3n and L4n correspond to the muscles controlling the distal interphalangeal (DIP) joint.
- V1, V2: Activations of neurons in the agonist and antagonist channels, respectively. Integrate excitatory and inhibitory inputs and calculate the ratio of opposing inputs.
- T1n, T2n, T3n, T4n: Normalized target lengths (TPV) of the muscles. T1n and T2n correspond to the muscles controlling the PIP joint, while T3n and T4n correspond to the muscles controlling the DIP joint.

#### 2.3.5. Learning and Adaptation

- Adjustment of gains and parameters: The various parameters and gains in the model could be adjusted through learning processes to optimize system performance using supervised or reinforcement learning algorithms.
- Adaptation of internal models: Internal models that predict the sensory consequences of motor commands and estimate the current state of the system are learned and adapted through experience, using prediction error as a learning signal.
- Learning of sensorimotor transformations: Transformations between sensory and motor spaces are learned through experience, forming and adjusting computational maps that relate sensory signals to appropriate motor commands.
- Cerebellar learning: An adaptive cerebellar loop is incorporated into the model to improve movement accuracy and coordination. The cerebellum learns to predict errors based on movement context and generate feedforward corrective commands.

**Figure 10:**
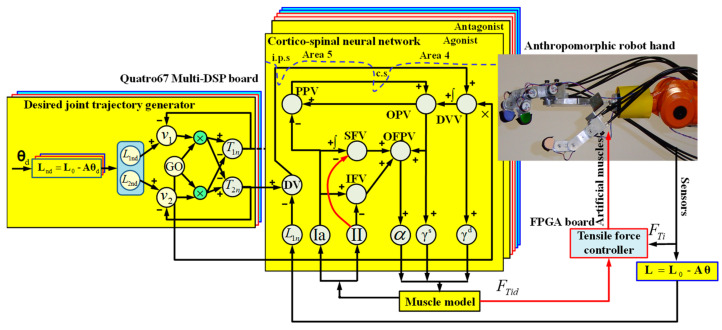
Architecture of the neurobiological control system for a robotic finger joint.

Once the model has learned and adapted, more precise, coordinated, and flexible movements are generated. The system’s capabilities are as follows:

- Afferents II: Secondary sensory fibers from muscle spindles primarily detect changes in muscle length.
- Generating smooth and well-controlled movement trajectories that adapt to different task demands and external conditions.
- Effectively compensating for perturbations and unexpected loads by dynamically adjusting motor commands based on sensory feedback.
- Adapting movements to changes in environmental dynamics or properties of the musculoskeletal system, such as fatigue or injuries.
- Improving motor performance through practice and refinement of internal models and sensorimotor transformations.
- In summary, the proposed corticospinal neural network model (Figure 10) integrates neurophysiological and computational knowledge to provide a biologically plausible framework for understanding voluntary motor control. The model highlights the importance of interaction between sensory and motor cortical areas, modulation of proprioceptive feedback, and dynamic compensation for generating precise and adaptive movements.

Incorporating learning and adaptation mechanisms into the model will further enhance its ability to capture the flexibility and robustness of the biological motor control system. As the model learns and adapts through experience, it is expected to generate increasingly natural and effective movements, even in the presence of perturbations and changes in the environment.

The corticospinal neural network model serves as a valuable tool for investigating the computational and neurophysiological principles underlying voluntary motor control and can provide insights into motor disorders and rehabilitation strategies. Furthermore, the principles embodied in the model can inspire the design of more adaptive and flexible robotic control systems.

In the future, it will be important to further validate and refine the model through experimental studies and comparisons with neurophysiological and behavioral data. The incorporation of additional anatomical and physiological details, such as recurrent connectivity and nonlinear neuronal dynamics, could improve the model’s ability to capture the complexity of the biological motor control system.

Moreover, integrating the model with other neural systems, such as the cerebellum, basal ganglia, and higher cortical areas, provides a more comprehensive view of how the brain generates and controls voluntary movements.

In conclusion, the proposed corticospinal neural network represents a significant advancement in our understanding of biological motor control and opens up new avenues for research in computational neuroscience, bioinspired robotics, and clinical applications. Through the continued integration of experimental and theoretical data, this approach promises to shed light on the fundamental principles underlying the nervous system’s remarkable ability to generate flexible and adaptive movements.

### 2.4. Hardware Implementation for the Neurobiological Control System

**Figure 11:**
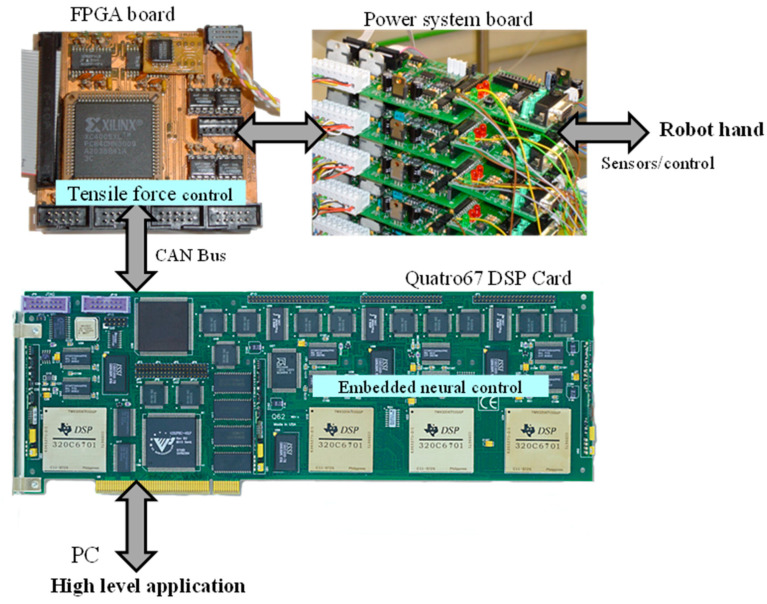
Electronic hardware of the mechatronic system of the anthropomorphic robot hand.

The hardware implemented for the robot hand, as shown in Figure 11, consists of four main components:

1. A PC for sensorimotor coordination of the robot hand.
2. A multi-DSP Quatro-67 board containing four TMS320C6701 digital signal processors (DSP), with the corticospinal neural control system for each finger implemented on a DSP.
3. Four Xilinx^®^ FPGA boards, each controlling one finger of the robot hand, implementing tendon traction force controllers, and performing sensor signal processing.
4. A CAN Bus interface board for communication between the Quatro67 board and the FPGA boards.

The user interfaces for the multi-DSP Quatro-67 and FPGA boards were created on a PC with an Intel^®^ Core™ i7-4770K processor and 8 GB of RAM. Dynamic Link Libraries (DLLs) provide transparent low-level and high-level calls for the user, and these calls are programmed in MS Visual C++ (Visual Studio 2019). 

**Figure 12:**
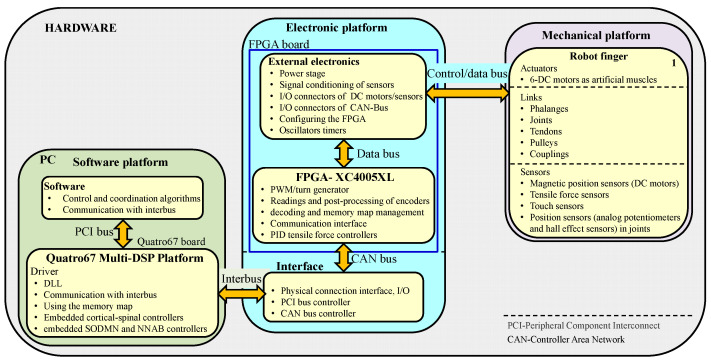
Functional structure of the mechatronic system of the anthropomorphic robot hand.

The hardware platform has an open system architecture because it allows access to the FPGA boards and the DSP processor board through DLLs, enabling communications with the hardware system and high-level system that are transparent to the user. From the software platform, through the system bus-level communication interface, it allows all the programming possibilities of the FPGA boards and configurations of the DSP processors to be implemented. Figure 12 shows the functional structure of the platforms that make up the mechatronic design of the anthropomorphic robotic hand.

On each Xilinx^®^ FPGA board, six PID traction force controllers with PWM (pulse width modulation) generation for Maxon^®^ DC micromotors have been implemented and tested. For this design, real-time execution on PID traction force controllers occurs at 5 KHz. 

The sensor signal conditioning circuits have implemented the following factors:

- Maxon^®^ digital magnetic encoders (Ø 13 mm, 16 counts per turn, 2 channels) used to measure the angular position of the micromotors, and the noise was limited by an analog low-pass filter with a cutoff frequency of 16 KHz.
- Traction force sensors (Burster^®^ 8417E subminiature load cell) used to measure the traction force of the tendons, and the sensor noise was limited with a fourth-order analog low-pass Butterworth filter at 1000 Hz.
- Precision potentiometers with continuous mechanical rotation (Vishay^®^ Spectrol Mod. 157S103MX) used to measure speed, direction of rotation, and angular position of the joints. The noise from these sensors was limited with a 100 Hz analog low-pass filter. As an auxiliary measure for the joint positions, Hall effect sensors (Honeywell^®^ SS490 series) were applied. The tendon lengths were determined through an indirect measurement.

**Figure 13:**
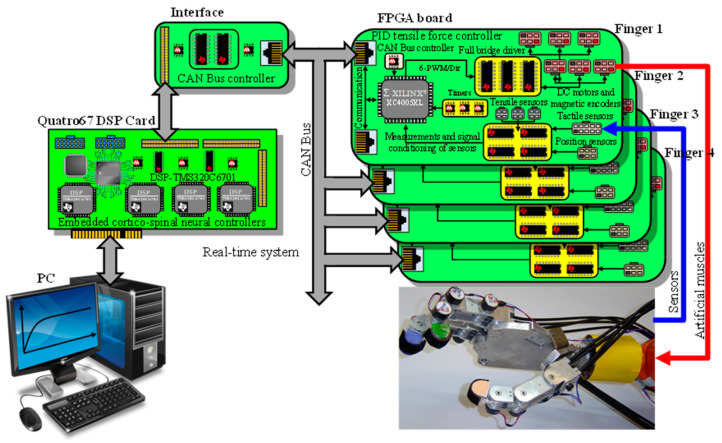
Implementation scheme of the neurobiological control system of the anthropomorphic robot hand.

The multi-DSP Quatro67 board is a standard 32-bit full-size peripheral component interconnect (PCI) board. Each TMS320C6701-TI^®^ processor features several on-chip peripherals including two 32-bit timers/counters, four flexible DMA channels, 1 Mbit of on-chip RAM, a dedicated host port interface (HPI), and a prioritized interrupt controller. The Quatro67 memory includes a 128 k × 32 asynchronous SRAM (ASRAM) region for bus mastering transfers and up to 16 M × 32 of 1-wait state synchronous DRAM (SDRAM) per processor. Each DSP on the Quatro67 board operates at 150 MHz and implements the neurobiological control system for the joint movement control of a robotic finger. This control system consists of three corticospinal neural network controllers with their trajectory generators. The Quatro67 board is hosted in a PCI slot of the PC, as illustrated in Figure 13.

### 2.5. Software Implementation for the Neurobiological Control System

The corticospinal neural circuits and their trajectory generators for voluntary joint movement control are programmed in C language, compiled, and downloaded onto the TMS320C6701 DSP, with real-time execution at 1 MHz. The fourth-order Runge–Kutta numerical integration method is used to solve the differential equations. 

The Quatro67 communication module sends and receives data to and from the communication module of the FPGA boards. 

The Quatro67 board must

- Establish communication with the FPGA boards.
- Send cortical network output signals (desired traction forces) to the FPGA boards in which the PID traction force controllers carry out the contraction of the artificial muscles.
- Receive the actual traction force, values of joint positions, tendon traction forces, angular positions of DC motors, and touch forces.

The PC monitors and implements a high-level sensor-motor coordination algorithm for the robot hand. This algorithm is based on studies in humans and animals that derived and inspired the SODMN and NNAB models for adaptive and reactive spatial navigation. In addition, the PC initiates the experiments, controls software execution, data logging, and selection of experimental parameters. 

The desired joint positions are sent from the base program of robot hand coordination to the DSP processor, where these signals are transformed between joint space and tendon space into the desired lengths of artificial muscles. These transformations are sent to the trajectory generators, smoothing the input signals to the corticospinal controllers through reference signals in the form of a target position vector. The corticospinal controllers generate alpha motor neuron signals as activation commands for artificial agonist and antagonist muscle models and receive feedback signals from the actual muscle spindles and actual muscle lengths. The muscle spindles calculate actual position and velocity error signals, where the primary spindle afferents (Ia) integrate these errors and the secondary spindle afferents (II) report position errors. 

The artificial muscle models generate the contractile dynamics of the artificial muscles to generate the forces in the tendons necessary to carry out or maintain a desired joint position and receive the actual tendon lengths and actual artificial muscle forces as feedback signals. The muscle lengths ($L_{i}$) are obtained by Equation (20).

(20) $$L=L_{0}-A\theta$$

The forces generated by the muscle models are sent as reference signals to the PID traction force controllers. These controllers are programmed on the FPGA board to maintain the tendons with the appropriate stiffness, as indicated by the corticospinal controllers, and receive feedback signals of the current forces originating in the artificial muscles. The PID controllers on the FPGA boards were programmed in VHDL language. 

**Figure 14:**
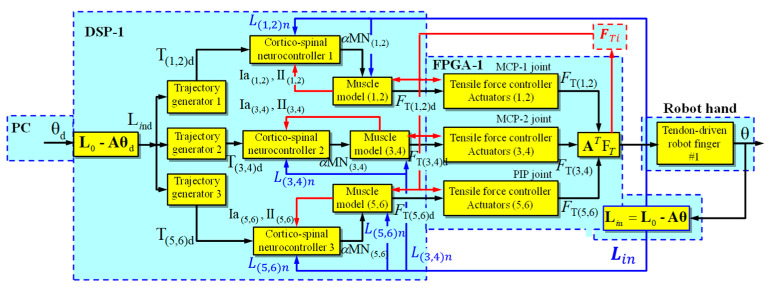
Simplified block diagram of the corticospinal neural control system of the three degrees of freedom robotic finger with a coupled joint. The subscripts *d* and *n* denote the terms desired and normalized, respectively.

Figure 14 shows the simplified block diagram of the control system implemented and programmed on a DSP processor and an FPGA board for each robotic finger.

### 2.6. Coordination of Complex Movement through the Proposed Corticospinal Algorithm

The proposed corticospinal controller is inspired by the organization and function of neuronal circuits in the biological nervous system, enabling it to generate coordinated and complex movements in the robotic hand. Coordination between the fingers, joints, and palm is achieved through the interaction of different neuronal modules within the controller’s architecture.

A set of learned movement primitives, similar to the muscle synergies observed in biological motor control, is utilized. These primitives represent spatiotemporal patterns of muscle activation that can be combined and scaled to generate a wide range of coordinated movements.

In the controller, the movement primitives are implemented as a weighted combination of force commands for the agonist and antagonist tendons of each joint. The selection and activation of these primitives are based on the desired task and available sensory information, allowing for flexible and adaptable generation of coordinated movements.

#### 2.6.1. Modulation of Impedance and Compliance

The corticospinal controller also incorporates mechanisms to dynamically modulate the impedance and compliance of the joints, emulating the adaptability of biological soft tissues. This is achieved by adjusting the feedback gains and stiffness parameters in the low-level controllers of each joint.

Impedance modulation allows the robotic hand to adapt to different interaction conditions, such as contact with rigid objects, and compensate for external perturbations. This translates into more stable grasping and robust manipulation, as the hand can adjust its compliance as needed.

#### 2.6.2. Sensorimotor Integration and Tactile Feedback

The coordination of complex movement in the corticospinal controller also relies on the tight integration of sensory and motor information. Proprioceptive data from position and force sensors in the joints are used to modulate control signals and adapt movements in real time.

Furthermore, tactile feedback from force sensors in the fingertips and phalanges is used to adjust grasping forces and detect slips or perturbations. This tactile information enables more precise and reactive control of the interaction between the hand and manipulated objects.

#### 2.6.3. Sensorimotor Coordination Algorithm for Finger Cooperation

The sensorimotor coordination algorithm implemented in the PC is based on the SODMN (Self-Organizing Dynamic Motor Network) and NNAB (Neural Network for Adaptive Behavior) models, which are derived from studies on adaptive and reactive spatial navigation in humans and animals.

This algorithm generates desired trajectories for each finger of the robotic hand, considering the position and orientation of the object to be grasped, the desired grasp shape, and the necessary synchronization between fingers. The process can be described in the following steps:

1. A perception of the object is created using information from visual sensors (cameras) and tactile sensors in the fingertips. The position, orientation, and approximate geometry of the object in the hand’s workspace are estimated.
2. An adaptive grasp is generated using movement primitives.
3. Desired trajectories are created for each finger involved in the grasp, defining waypoints and arrival times. These trajectories consider the kinematics and dynamics of the hand, as well as collision constraints and joint limits.
4. The finger trajectories are temporally synchronized to ensure coordinated movement during the grasp. This involves adjusting start and end times, as well as relative velocities between fingers.
5. The desired trajectories are sent to the corticospinal controllers of each finger, which generate motor commands for the actuators, considering the system dynamics and sensory feedback.
6. During the execution of the grasp, tactile and proprioceptive sensor signals are continuously monitored. If significant deviations from the desired trajectories or changes in object conditions are detected, compensation commands are generated, and the trajectories are adapted in real time.

This algorithm allows for smooth and effective coordination between the fingers of the robotic hand during manipulation tasks, emulating the dexterity and adaptability observed in human hands. The combination of high-level planning and reactive control based on sensory feedback enables the robust execution of grasps in the presence of uncertainties and perturbations.

## 3. Results

**Figure 15:**
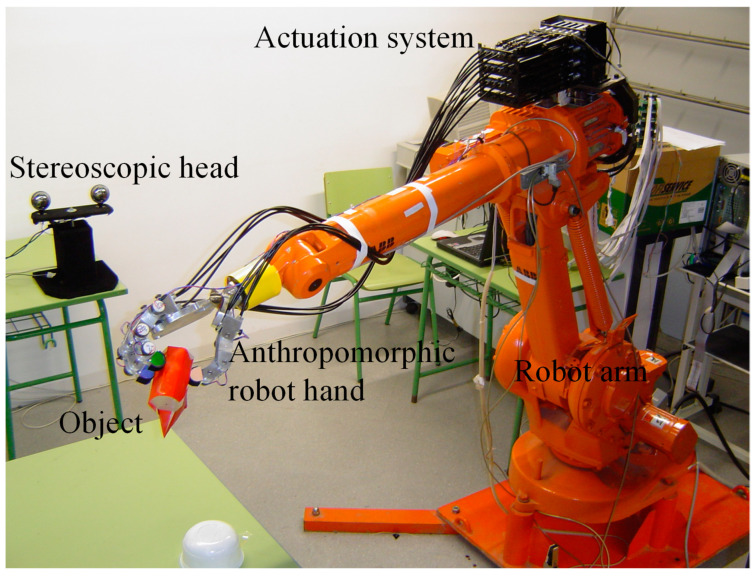
Experimental platform for reaching and grasping applications.

The proposed corticospinal neural control system was implemented on a tendon-driven multi-fingered anthropomorphic robotic hand to control voluntary joint movements. The experiments were carried out on a robotic platform consisting of a 5 degrees of freedom (DoF) stereoscopic head, a 6 DoF robotic arm, and an anthropomorphic hand (Figure 15). The main objective was to evaluate the controller’s performance in reaching and grasping tasks, as well as its ability to generate human-like joint movement trajectories, exhibiting robustness to perturbations and adaptability to different conditions.

### 3.1. System Response to Various Inputs

**Figure 16:**
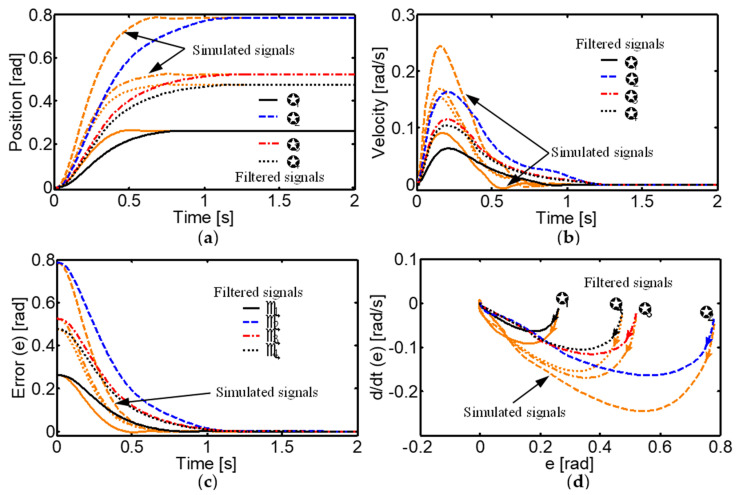
Simulated and real neural control system responses to a desired step input into the joints with θ_1d_ = 15°, θ_2d_ = 45°, θ_3d_ = 30°, and θ_4d_ = (r_13_/r_15_) θ_3d_. (**a**) Joint positions; (**b**) speed profiles; (**c**) convergence of error; (**d**) phase plane of the neural control system.

A comprehensive analysis of the control system response to various inputs was performed, including step, square wave, sinusoidal, and exponential trajectories. For step inputs, specific desired joint angles were set for the metacarpophalangeal (MCP_1_ and MCP_2_), proximal interphalangeal (PIP), and distal interphalangeal (DIP) joints. The results showed that the system quickly reached the desired positions without overshooting. In particular, the settling times for the MCP_1_, MCP_2_, PIP, and DIP joints were around 0.6 s, 0.7 s, 0.8 s, and 0.8 s in the simulations, and 0.8 s, 1.2 s, 1.3 s, and 1.3 s in the experiments, respectively. Additionally, the steady-state errors were extremely low, with RMSE values of 4 × 10^−8^, 1.3 × 10^−6^, 3.3 × 10^−6^, and 3 × 10^−6^ radians in the simulations, and 1 × 10^−6^, 3 × 10^−5^, 1.8 × 10^−5^ and 1.6 × 10^−5^ radians in the experiments for the MCP_1_, MCP_2_, PIP, and DIP joints, respectively (Figure 16). These results demonstrate the accuracy and speed of the controller in reaching the desired positions.

**Figure 17:**
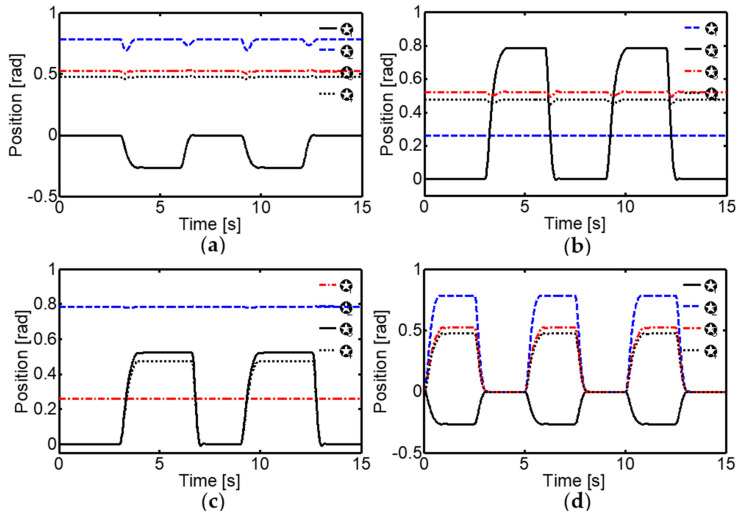
Joint movement sequence of the four-link robot finger for stepping. (**a**) Movement variation in the MCP_1_ joint with angular step inputs from $\theta_{1d}=0{}^{\circ}\;to\;-15{}^{\circ}$, while the MCP_2_, PID, and DIP joints remain constant at a desired position. (**b**) Movement trajectory in the MCP_2_ joint with step inputs from $\theta_{2d}=0{}^{\circ}\;to\;45{}^{\circ}$, while the MCP_1_, PIP, and DIP joints remain constant at a desired position. (**c**) Movement trajectory in the PIP joint using setpoint changes at intervals of $\theta_{3d}=0{}^{\circ}\;to\;30{}^{\circ}$, while the MCP_1_ and MCP_2_ joints remain constant at a desired position. (**d**) Movement sequence of the MCP_1_, MCP_2_, and PIP joints at intervals of $\theta_{1d}=0{}^{\circ}\;to\;-15{}^{\circ}$, $\theta_{2d}=0{}^{\circ}\;to\;45{}^{\circ}$ and $\theta_{3d}=0{}^{\circ}\;to\;30{}^{\circ}$, respectively.

Regarding square wave trajectories, the system’s ability to follow sudden changes in the desired joint positions was thoroughly evaluated. It was observed that the corticospinal neural controller was highly robust and adaptable to the determined alterations in the joints, quickly compensating for the changes and maintaining minimal errors (Figure 17). This was achieved due to the controller’s ability to adjust tendon stiffness and generate high and low gain forces according to the task requirements. For example, when position changes occurred in the MCP_1_ joint, the MCP_2_ joint was perturbed with a variation of −0.08 radians for 0.6 s, while the PIP and DIP joints were perturbed with small oscillations between 17 × 10^−3^ and −1.2 × 10^−3^ radians for 0.8 s. However, the corticospinal controller quickly compensated for these perturbations and maintained the desired joint positions.

Furthermore, the performance of the control system for tracking sinusoidal and exponential trajectories was studied in detail. The desired joint trajectories were defined as follows:

(21) $$\theta_{1d}=15\pi /180sin\left(0.05t-\pi /2\right)$$

(22) $$\theta_{2d}=45\pi /180sin\left(0.05t\right)$$

(23) $$\theta_{3d}=15\pi /180sin\left(0.05t-\pi /2\right)$$

(24) $$\theta_{4d}=\left(r_{13}/r_{15}\right){\;\theta }_{3d}$$

with a variation of the parameter $g^{\left(0\right)}=15$ in the GO signals.

**Figure 18:**
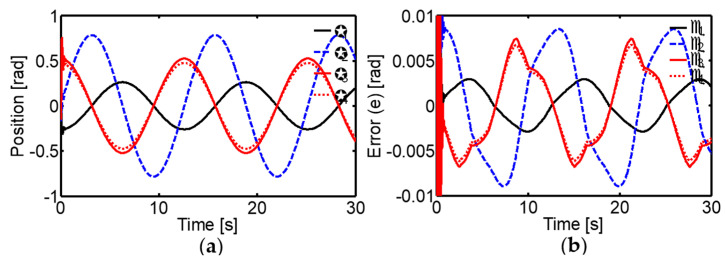
Performance of the biologically inspired neural network for trajectory tracking control. (**a**) Joint positions. (**b**) Tracking errors.

The results showed exceptionally accurate tracking of the desired trajectories, with very low tracking errors (Figure 18). This is due to the controller’s ability to generate bell-shaped asymmetric velocity profiles similar to those observed in human movements.

### 3.2. Stability and Robustness Analysis

The stability of the neural control system was thoroughly analyzed using phase planes for different initial conditions (Table 4). The phase planes revealed a stable focus for all initial conditions, implying that both the error e(t) and its derivative ė(t) converge to zero. 

**Figure 19:**
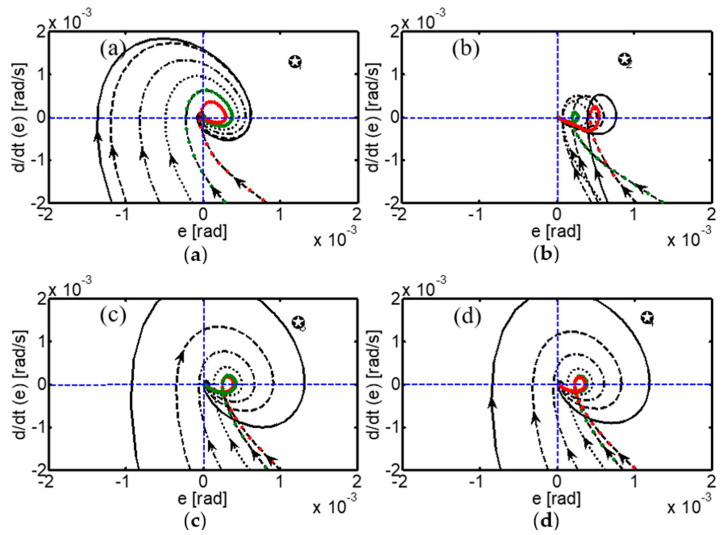
Asymptotically stable convergence of the biologically inspired control system. Phase plane of the neural control system for (**a**) MCP_1_, (**b**) MCP_2_, (**c**) PIP, and (**d**) DIP joints.

The different initial points of the control system are stable clockwise spiral points and the orbits move in the direction of the origin, demonstrating that the system is uniformly asymptotically stable (Figure 19). This rigorous stability analysis provides a solid theoretical foundation for the controller’s application in real robotic systems, ensuring stable and predictable behavior even under different initial conditions.

**Figure 20:**
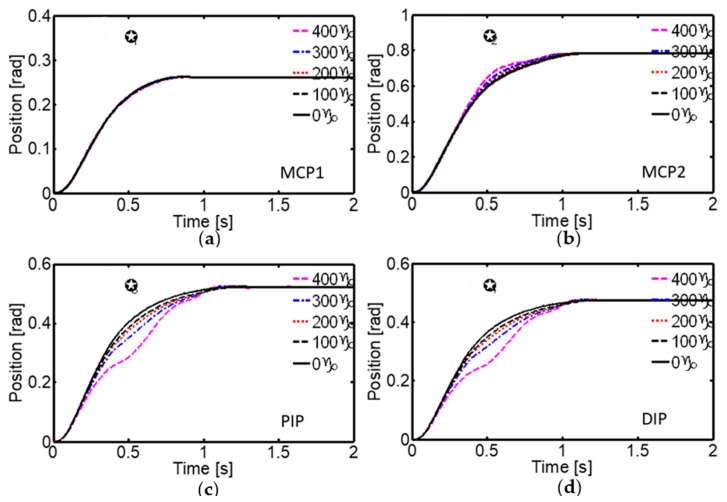
System response to external disturbances. The step responses of the MCP_1_, MCP_2_, PIP, and DIP joints to the mass variation of link 4 are shown in (**a**), (**b**), (**c**), and (**d**), respectively. The desired angular positions were $\theta_{1d}=15{}^{\circ}$, $\theta_{2d}=45{}^{\circ}$, $\theta_{3d}=30{}^{\circ}$ and ${\;\theta }_{4d}=\left(r_{13}/r_{15}\right){\;\theta }_{3d}$.

One of the key aspects evaluated was the robustness of the control system against external disturbances, such as mass variations in the links and suddenly applied loads. Extensive experiments were conducted by adding proportional masses to the links from the rest position, and a gradual increase in settling time was observed as the mass increased. In particular, for an additional load of 400 g on link 4, the settling times reached maximum values of 1.35 s, 1.45 s, 1.55 s, and 1.55 s for the MCP_1_, MCP_2_, PIP, and DIP joints, respectively (Figure 20, Table 5).

Despite this increase in settling time, the neurobiological control system effectively compensated for these disturbances, maintaining joint positions and adjusting torques appropriately.

**Figure 21:**
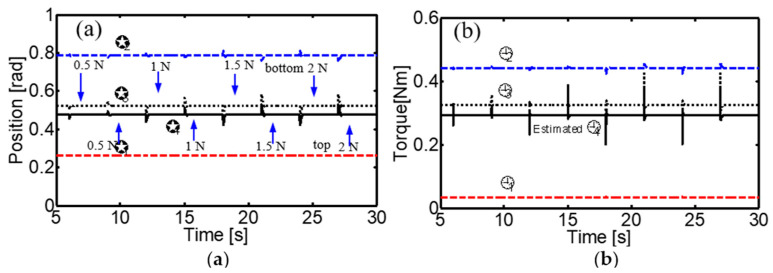
Performance of the neural control system for load disturbances at the fingertip. Load disturbances were applied in opposite directions on the DIP joint link with loads of 0.5 N, 1 N, 1.5 N, and 2 N. (**a**) Joint positions, (**b**) joint torques.

Furthermore, sudden loads of different magnitudes (0.5 N, 1 N, 1.5 N, and 2 N) were applied in opposite directions while the joints remained in the desired position, and the controller demonstrated excellent ability to compensate for these disturbances, maintaining joint positions and adjusting torques accordingly (Figure 21).

**Figure 22:**
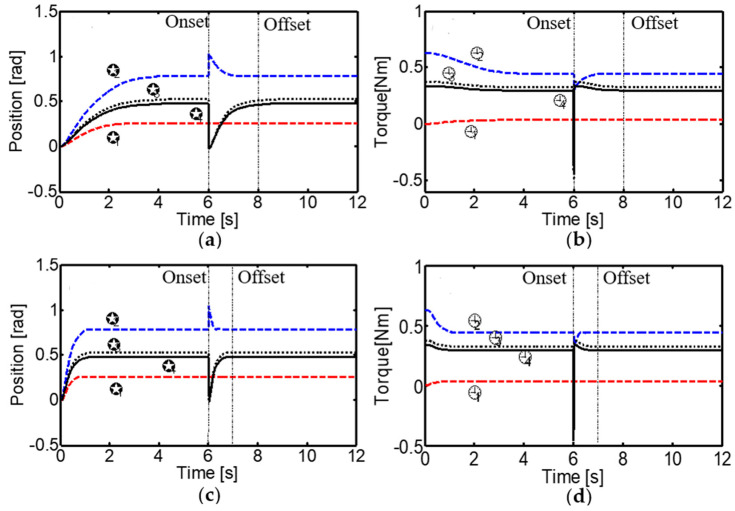
Performance of the neural control system under a static load disturbance of 15 N at the fingertip. Dashed vertical lines indicate the onset and offset of the load. The active stiffness in (**a**,**c**) is implemented by setting $g^{\left(0\right)}=0.5$ and $g^{\left(0\right)}=2$, respectively. The torques in (**b**,**d**) are calculated from $F_{Ti}-F_{Tj}$.

The system’s performance under a significant static load disturbance of 15 N at the fingertip was also thoroughly evaluated. The corticospinal controller compensated for the disturbance by adjusting tendon stiffness through the GO signal. It was shown that by increasing the *g*(0) value of the GO signal from 0.5 to 2, the system compensates for load disturbances more quickly, increasing tendon stiffness and improving the response time (Figure 22). This highlights the controller’s ability to adapt its behavior based on task demands and external conditions, a skill that is crucial for robust and effective interaction with the environment.

### 3.3. Reaching and Grasping Tasks

To evaluate the system’s capability in reaching and grasping tasks, extensive experiments were conducted with objects of different shapes and sizes. Two movement sequences were defined: Sequence-1 for reaching and grasping a cylindrical object, and Sequence-2 for a regular hexagonal object.

**Figure 23:**
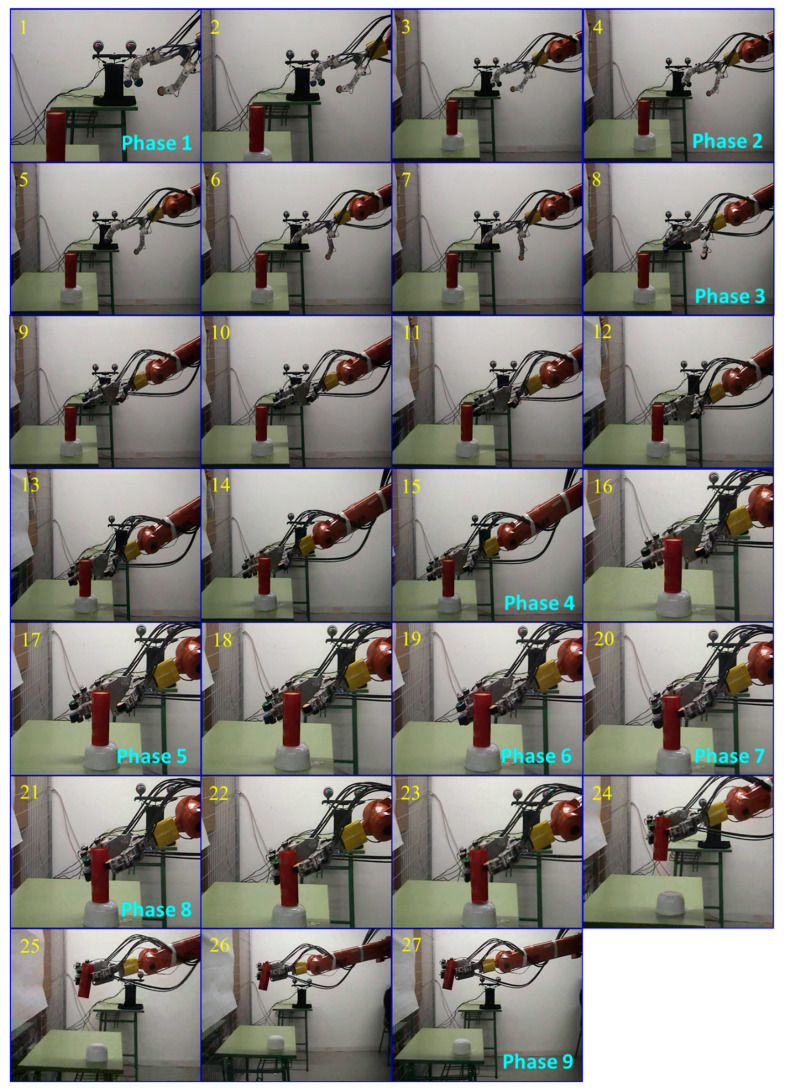
Sequence-1 of reaching and grasping an object.

Figure 23 shows Sequence-1 of the reaching and grasping process for a cylindrical object with a diameter of 5 cm and a length of 12 cm. The sequence consists of nine phases, which are as follows:

1. Initiation of Sequence-1. t = 4 s, frame = 1.
2. Pre-grasping of the robotic hand. t = 5 s, frame = 4.
3. Wrist rotation and reaching towards the object. t = 6 s, frame = 8.
4. Orientation and approach of the robotic hand to the object. t = 9 s, frame = 15.
5. Initiation of the grasping process. t = 12 s, frame = 17.
6. Contact established with the object. t = 13 s, frame = 19.
7. Stable grasp achieved. t = 14 s, frame = 20.
8. Initiation of object lifting with a stable grasp. t = 15 s, frame = 21.
9. Completion of Sequence-1. t = 18 s, frame = 27.

Sequence-2 involves the reaching and grasping of a regular hexagonal object with a diameter of 5 cm and a length of 10 cm. This sequence follows the same steps as Sequence-1. 

**Figure 24:**
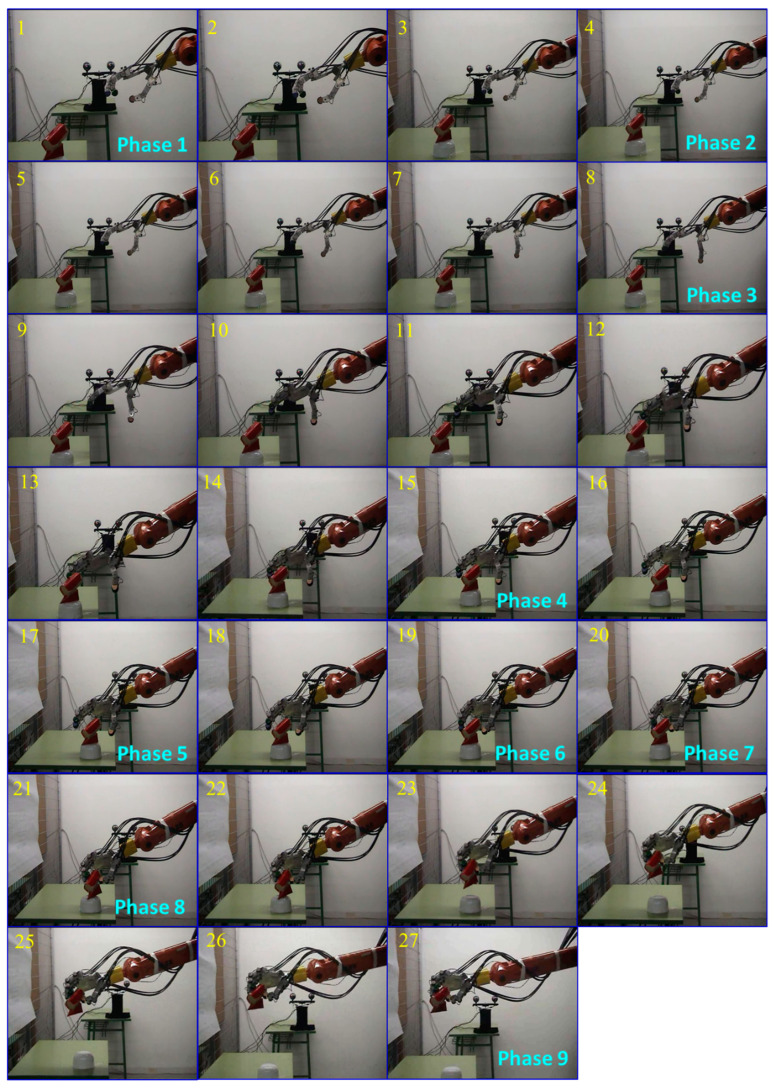
Sequence-2 of reaching and grasping an object.

Figure 24 illustrates the nine main phases of reaching and grasping the hexagonal-shaped object. Sequence-2 begins at frame 1, where the stereoscopic vision system identifies the location and orientation of the object within the robot arm–hand workspace. The opening of the hand as a pre-grasping step starts at frame 4. Subsequently, the approximation and orientation of the robot wrist-hand towards the target is generated, as shown in frame 8. After the initial approximation, the final positioning of the hand with respect to the target is initiated, as seen in frame 15. The closing and grasping phase of the object commences at frame 17. In frame 19, the robotic hand makes its first contact with the object, followed by a stable grasp recorded in frame 20. Frame 21 marks the beginning of the object lifting phase with a stable grip maintained throughout the movement. The sequence concludes in frame 27.

The results show that the robotic hand was able to adopt the necessary postures for each phase, represented by the precise joint angles of the fingers (Table 6), and perform a stable grasp of the objects.

This demonstrates the versatility and adaptability of the controller for different manipulation tasks, enabling effective interaction with objects of various characteristics.

The joint angles of the fingers during the sequences were calculated using a mathematical expression that considers the contribution of each angular joint of a specific finger.

**Figure 25:**
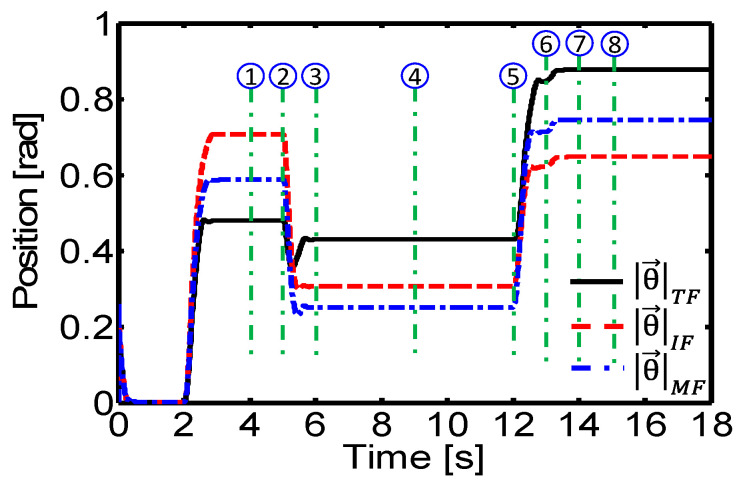
Experimental result. Joint angles of the robot hand’s fingers in Sequence-1.

**Figure 26:**
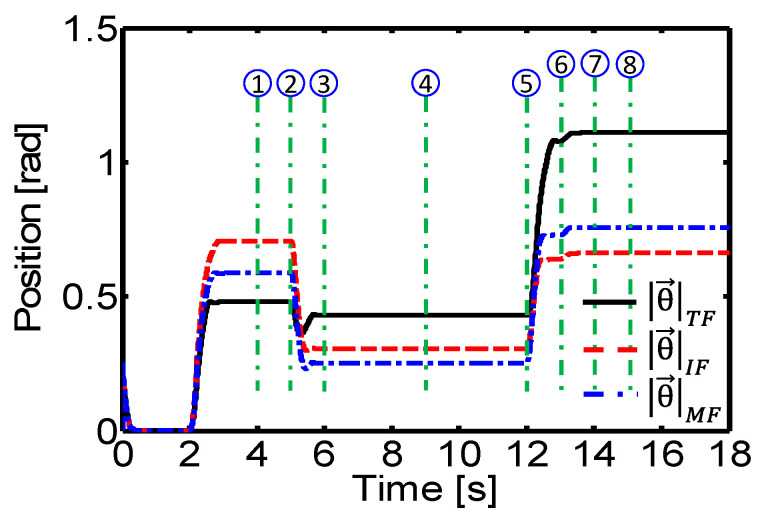
Experimental result. Joint angles of the robot hand finger in Sequence-2.

The expression used was

(25) $${\left|\vec{\theta }\right|}_{j}=\sqrt{\sum_{i=1}^{4}\theta_{i}^{2}}$$

where *i* represents the angular joint of a finger and *j* = {TF, IF, MF}, TF is the thumb, IF is the index finger, and MF is the middle finger (Figure 25 and Figure 26). This quantitative analysis allows an objective evaluation of the controller’s performance and provides valuable information for future adjustments and improvements, as it allows comparison of the joint angles generated by the controller with the desired angles for each manipulation task.

**Figure 27:**
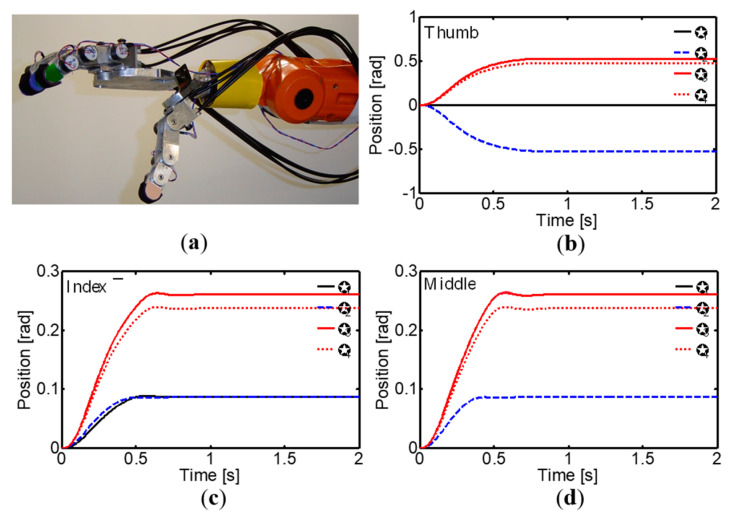
(**a**) Posture I of the anthropomorphic robot hand. (**b**) Thumb joint angles. (**c**) Index finger joint angles. (**d**) Middle finger joint angles.

**Figure 28:**
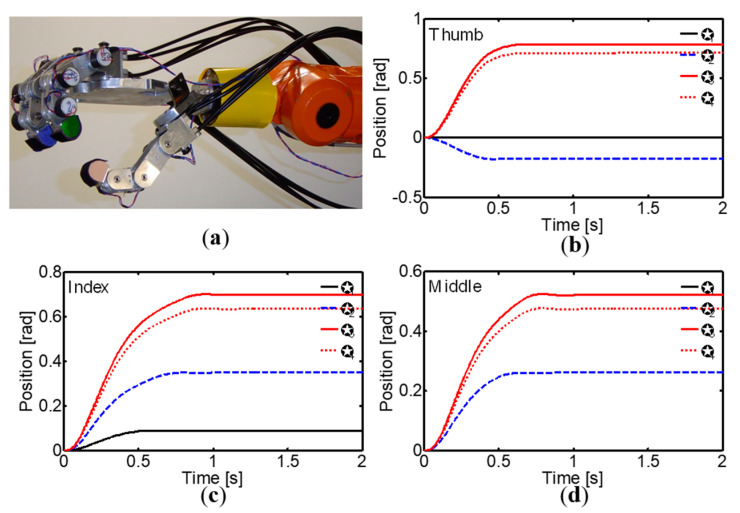
(**a**) Posture II of the anthropomorphic robot hand. (**b**) Thumb joint angles. (**c**) Index finger joint angles. (**d**) Middle finger joint angles.

The versatility and adaptability of the corticospinal controller with regard to generating specific postures of the robotic hand during reaching and grasping tasks is highlighted in Figure 27 and Figure 28. These postures, defined by the desired joint angles for each finger, demonstrate the controller’s ability to adjust the hand configuration according to the requirements of specific tasks. In posture I (Figure 27), the joint angles generated by the controller allow for an adequate hand opening and orientation for pre-grasping and reaching for the object, while in posture II (Figure 28), the joint angles facilitate a stable grasp of the object. These figures highlight how the controller can adapt to different hand postures, which is fundamental for successful manipulation of objects with various shapes and sizes. Moreover, the ability to generate and maintain these specific postures during the different phases of reaching and grasping tasks demonstrates the robustness and stability of the corticospinal controller in realistic manipulation environments.

### 3.4. Balance between Active and Passive Movement

The experimental results demonstrated that the corticospinal controller allows a balance between active and passive movement responses according to internal and external demands, which are controlled by two separate gating operations. The GO signal controls the speed of voluntary movement and the effort exerted against obstructions, while the SFV integration rate (parameter h) controls the gain of load compensation. During normal operation, parameter h is set to a low value, resulting in low-gain load compensation. However, during tasks that demand high force generation, such as lifting an extra load or when loads are suddenly applied, parameter h increases, resulting in high-gain load compensation. This dynamic balance between active and passive movement makes the system robust against external disturbances like sudden passive and active loads, allowing for rapid and effective adaptation to different conditions.

### 3.5. Generation of High and Low Gain Forces

It was observed that the controller is capable of generating high and low gain forces, adapting to tasks that demand different levels of force. During normal operation, force generation is low gain, but it significantly increases during tasks that require high force generation, such as lifting an extra load or when loads are suddenly applied. This is achieved through muscle-specific gains in the SFV integration (parameter k), which allow dynamic adaptation to the force demands of the task at hand.

### 3.6. Fast and Slow Movements

Another notable aspect is the controller’s ability to allow for fast and slow movements of robotic systems. During fast movements, the system reduces the sensitivity of muscle spindles to stretch by decreasing the activation of static gamma motor neurons, switching from a feedback position controller during slow movements to a feedforward trajectory generator with feedback velocity compensation during fast movements. This enables a smooth and effective transition between different control modes according to the desired movement speed.

### 3.7. Simplified Properties of the Model

It is important to highlight that the proposed model presents several simplified properties of the motor control system. The neural computations involve populations with pairs of neurons, each corresponding to a member of an agonist–antagonist pair, which allows us to understand the broad functional roles played by different cell classes. Furthermore, two distributed schemes are used in the corticospinal network: the population vector for directional tuning in the motor and sensorimotor cortex, and the size principle-like recruitment scheme of alpha motor neurons. These schemes were manually implemented in the proposed network but could be introduced through cerebellar learning in the neural model to modify velocity commands and overcome the inherent limitations of spinal tracking feedback control. These simplifications allow for a more efficient implementation of the controller while maintaining a high degree of biological plausibility.

## 4. Discussion

The experimental results demonstrate that the proposed corticospinal neural control system is capable of generating precise, robust, and adaptive movements in a tendon-driven anthropomorphic robotic hand. The controller exhibits several key properties of biological motor control, such as bell-shaped asymmetric velocity profiles [11], dynamic compensation for disturbances [12], and adaptability to different task demands [17]. These findings align with previous studies that have highlighted the importance of these aspects in human motor control and have sought to incorporate them into bioinspired robotic systems [36,37].

A notable contribution of this work is the controller’s ability to balance active and passive movement responses according to internal and external demands. This approach, which was achieved through two separate gating operations (GO signal and the SFV integration rate), allows for rapid and effective adaptation to different conditions, enhancing the system’s robustness to external perturbations [24]. This feature resembles the human nervous system’s ability to dynamically modulate motor control in response to changes in the environment or task demands [38].

Furthermore, the controller’s ability to generate high and low gain forces and allow for fast and slow movements demonstrates its versatility and biological plausibility. These characteristics are particularly relevant for manipulation tasks involving variable interactions with the environment [19] and reflect the flexibility of human motor control [39].

However, it is important to acknowledge some of the limitations of the study. The proposed model incorporates several simplifications, such as the use of populations with pairs of neurons corresponding to an agonist–antagonist pair and the manual implementation of distributed schemes in the corticospinal network. While these simplifications enable a more efficient implementation of the controller, they may not capture the full complexity and nonlinearity of biological neural systems [32]. Future iterations of the model could seek to incorporate additional anatomical and physiological details to further enhance its biological plausibility.

Another limitation is that the experiments were conducted in a controlled laboratory setting with a limited set of reaching and grasping tasks. While these experiments demonstrated the controller’s versatility and adaptability, they may not capture the full range of challenges encountered in real-world environments. Future validations could involve a broader range of manipulation tasks and environmental conditions to more thoroughly assess the robustness and generalization of the proposed approach.

Despite these limitations, the presented findings have significant implications for both our theoretical understanding of biological motor control and practical applications in robotics and assistive technologies. From a theoretical perspective, this work demonstrates how principles of biological motor control, such as modulation of proprioceptive feedback and dynamic compensation, can be implemented in a computational framework [33]. This could inform the development of more sophisticated and biologically plausible neural models of motor control.

In terms of practical applications, the proposed approach could be incorporated into the design of advanced prostheses and autonomous robotic systems. By more closely emulating the capabilities of human motor control, these systems could achieve greater dexterity, adaptability, and robustness [40]. This could have significant implications for domains such as service robotics, manufacturing, and healthcare.

To advance this line of research, there are several promising directions to follow, and the manipulation of deformable and soft objects is one of them. The results presented in this study demonstrate the capability of the anthropomorphic robotic hand and the corticospinal controller to effectively manipulate rigid objects with different geometries. However, we recognize that many practical applications of robotic manipulation also require interaction with deformable and soft objects, such as tissues, food, or flexible components.

To address this challenge, we propose extending our approach to include the manipulation of non-rigid objects. This is an important direction for future research and will involve several key aspects:

1. Modeling deformable objects: Developing efficient computational models that capture the dynamics and mechanical properties of deformable objects, such as elasticity, plasticity, and viscoelasticity. These models will allow us to predict the object’s deformation under the action of forces applied by the robotic hand.
2. Advanced tactile sensing: Incorporating more sophisticated tactile sensors, such as pressure sensor arrays or capacitive tactile sensors, that can measure pressure distribution and detect local deformations on the object’s surface. This enriched sensory information will enable more precise force control and adaptation to object deformation during manipulation.
3. Adaptive control strategies: Extending the corticospinal controller to incorporate adaptive control strategies that can adjust grasping forces and trajectories based on object deformation and estimated properties. This may involve the use of reinforcement learning techniques or model-based predictive control to optimize the manipulation of deformable objects.
4. Experimental validation: Conducting extensive experiments with a variety of deformable and soft objects, such as sponges, rubber balls, fabrics, and simulated biological materials. These experiments will evaluate the ability of the robotic hand and controller to adapt to different levels of deformation, maintain stable grasps, and manipulate the objects in a controlled manner.

By addressing these factors, we aim to significantly expand the manipulation capabilities of our anthropomorphic robotic hand and make it more applicable to a wider range of real-world tasks. The successful manipulation of deformable and soft objects would represent a major step forward in the development of versatile and adaptable robotic systems, with potential applications in fields such as service robotics, food handling, robotic surgery, and human–robot interaction.

Finally, continuous validation and refinement of the model through experimental studies and comparisons with neurophysiological and behavioral data will be essential [33]. This could involve interdisciplinary collaborations with neuroscientists to identify key biological constraints and refine the computational components of the model.

In conclusion, this study presents a novel corticospinal neural control system that successfully achieves robust and adaptable motor control in an anthropomorphic robotic hand. By integrating insights from neuroscience and control theory, this work contributes to both our fundamental understanding of biological motor control and the development of advanced robotic systems. While challenges remain, the promising results and diverse directions for future research highlight the potential of this approach to drive significant advances in bioinspired robotics, assistive technologies, and our understanding of the brain itself.

## 5. Conclusions

In this work, we present a novel corticospinal control algorithm for tendon-driven anthropomorphic robotic hands. The proposed algorithm is inspired by the organizational principles and function of neural circuits in the biological nervous system, and aims to generate dexterous, coordinated, and adaptive movements in multi-fingered robotic systems.

The main contributions and improvements of the corticospinal algorithm are as follows:

1. Generation of human-like movement profiles: The controller is capable of generating smooth trajectories and asymmetric bell-shaped velocity profiles similar to those observed in biological movements. This is achieved through the integration of movement primitives and the modulation of control signals based on sensory feedback.
2. Adaptability to disturbances and changes in the environment: The algorithm incorporates mechanisms for impedance modulation and adjustment of feedback gains that allow the robotic hand to adapt to different interaction conditions and compensate for external disturbances. This results in more stable grasping and more robust manipulation.
3. Coordination and synchronization of multi-finger movements: The controller uses movement primitives and coordination rules inspired by biological muscle synergies to generate coordinated and synchronized movements between fingers and joints. This enables the execution of complex and dexterous manipulation tasks.
4. Experience-based learning and optimization: The algorithm includes reinforcement learning and optimization mechanisms that allow controller parameters to be adjusted based on experience and task performance. This enables adaptation to new situations and continuous performance improvement over time.

The integration of the corticospinal algorithm with the tendon-driven anthropomorphic robotic hand system has proven to be successful and effective. The experiments conducted, which encompassed grasping and manipulation tasks with various objects, have validated the controller’s ability to generate dexterous, adaptive, and robust movements.

However, we also acknowledge some challenges and future directions to further improve the performance and applicability of the system:

1. Scalability to more complex robotic systems: It will be necessary to investigate the extension of the corticospinal algorithm to robotic hands with a greater number of fingers and degrees of freedom, as well as its integration with arm and full-body systems for more advanced manipulation tasks.
2. Improvements in computational efficiency: Optimizing the implementation of the algorithm and exploring parallelization and distributed computing techniques to enable more efficient real-time control, especially as system complexity increases.
3. Online learning and adaptation: Developing methods for continuous learning and online adaptation of the controller, allowing the system to improve its performance during operation and adapt to changes in the environment or task without requiring extensive retraining.
4. Validation in real-world environments and applications: Conducting thorough testing of the system in unstructured environments and practical applications, such as deformable object manipulation, human–robot interaction, and operation under variable and unpredictable conditions.

In conclusion, the proposed corticospinal control algorithm represents a significant advance in the dexterous and adaptive control of tendon-driven anthropomorphic robotic hands. The integration of biological principles and control theory has proven to be a promising approach to achieving human-like performance in complex robotic systems. We hope that this work lays the foundation for future developments in the field of robotic manipulation and contributes to the advancement of more capable, versatile, and autonomous robots.

## Figures and Tables

**Table 1:** Technical characteristics of the anthropomorphic robotic hand “Cervantes’s Hand”.

The anthropomorphic hand consists of a palm, 3 fingers and a thumb, has 16 DoF, 24 DC linear actuators, 12 tactile FSR sensors, 16 Hall-effect position sensors, and 24 strain sensors. The robot hand’s dimensions are 269(L) × 109(W) × 38(H) mm. The manufacture material is the 3000-group aluminum with a proportion of 1.82 manganese (Mn).			
Robot hand weight:	1.409 kg	Hand support weight:	0.053 kg
Palm weight:	0.328 kg		
Finger weight:	0.240 kg		
Thumb weight:	0.307 kg		
Dimensions of the robot hand			
Robot hand:	269(L) × 109(W) × 38(H) mm		
Palm of the robot hand:	135(L) × 109(W) × 12(H) mm		
Fingers and thumb:	158(L) × 27(W) × 27(H) mm		
Distance between finger joints			
MCP_1_ and MCP_2_ joints:	25 mm		
MCP_2_ and PIP joints:	53 mm		
PIP and DIP joints:	33 mm		
DIP joint and fingertip:	36 mm		
Dimensions of the finger phalanges			
MCP_1_ phalange:	45(L) × 33(W) × 33(H) mm		
MCP_2_ phalange:	77.5(L) × 30(W) × 25(H) mm		
PIP phalange:	57(L) × 23(W) × 24(H) mm		
DIP phalange:	47.5(L) × 20(W) × 24(H) mm		
Joint pulley diameters			
MCP_1_, MCP_2_, PIP and DIP joints:		⌀22 mm	
PIP joint:		⌀20 mm (⌀_13_ and ⌀_14_)	
L = Length, W = Width, and H = Height.			

**Table 2:** Technical characteristics of anthropomorphic robot fingers.

Anthropomorphic fingers have 4 DoF with 3 independent jointa, 6 DC linear actuators, 3 tactile FSR sensors, 4 position Hall-effect sensors, and 6 strain sensors. The robot’s finger dimensions are 158 (L) × 26 (W) × 26 (H) mm. The manufacture material is the 3000-group aluminum with proportion of 1.82 manganese (Mn).	
DoF Independent joints: MCP_1_ joint, MCP_2_ joint, and PIP-Joint. Coupled joint: DIP Joint with PIP-joint.	
Motions PIP and DIP joints: flexion–extension. MCP_1_ joint: abduction–adduction. MCP_2_: flexion–extension.	
Range of motion Abduction–adduction: ± 30° Flexion: 90° (all joints) Extension: 13° (MCP_2_ joint), 11° (PIP joint), and 6° (DIP joint)	
Sensors Tactile sensor: Force sensor resistors (FSR no. 402). Position sensor: Hall-effect sensor and potentiometers (initial phase). Force/tensile sensor: strain sensor (0–500 N).	
Actuator type Maxon^®^ DC motor Power rating: 3.2 W Nominal voltage: 24 V No load speed: 7250 rpm. Stall torque: 17 mNm. Max. continuous current: 172 mA.	Tendons Stainless-steel braided wire coated with nylon (AFW-060, 27 kg, ∅ = 0.81 mm). Tendons into pre-lubricated bicycle cable sheath (width = 4.5 mm, housing diameter = 4 mm).

**Table 3:** Denavit–Hartenberg parameters for the anthropomorphic robotic finger.

Joint	θ (rad)	d (mm)	a (mm)	α (rad)
**MCP_1_**	θ1	0	25	π/2
**MCP_2_**	θ2	0	53	0
**PIP**	θ3	0	33	0
**DIP**	θ4	0	36	0

**Table 4:** Initial conditions for phase portraits.

Angles/Conditions	*c*_1_	*c*_2_	*c*_3_	*c*_4_	*c*_5_	*c*_6_
${\mathrm{MCP}}_{1}\;(\theta_{1d}$)	5	10	15	20	25	30
${\mathrm{MCP}}_{2}\;(\theta_{2d}$)	10	15	20	25	30	35
$\mathrm{PIP}\;(\theta_{3d}$)	15	20	25	30	35	40
$\mathrm{DIP}\;(\theta_{4d}$)	$\left(r_{13}/r_{15}\right)\theta_{3d}$					

*c_i_* represents the initial conditions. $r_{13}=10\;mm$ and $r_{15}=11\;mm$.

**Table 5:** Settling time of the proposed neural system under load variation.

Loads in the Link 4	Settling Time (Seconds)			
	$\theta_{1}$	$\theta_{2}$	$\theta_{3}$	$\theta_{4}$
0 g	0.8	1.2	1.3	1.3
100 g	1	1.25	1.35	1.35
200 g	1.2	1.3	1.45	1.45
300 g	1.3	1.4	1.5	1.5
400 g	1.35	1.45	1.55	1.55

**Table 6:** Joint angles formed in the fingers of the robotic hand during reaching and grasping of an object.

	Start	Pre-grasping, Orientation, and Reaching	Grasping
Phases	1	2–4	5–8
Finger/angles	$\left(\theta_{1},\theta_{2},\theta_{3},\theta_{4}\right)$		
		Sequence-1	
TF	(15,0,20,18.18)	(5, −20,10,9.09)	(10, −5,35,31.82)
IF	(10,20,15,13.64)	(10, −5,10,9.09)	(10, −5,25,22.73)
MF	(0,20,20,18.18)	(0, −5,10,9.09)	(0, −5,30,27.27)
		Sequence-2	
TF	(5,0,20,18.18)	(5, −20,10,9.09)	(10, −5,45,40.91)
IF	(10,20,25,22.73)	(10, −5,10,9.09)	(10, −10,25,22.73)
MF	(0,20,20,18.18)	(0, −5,10,9.09)	(0, −10,30,27.27)

## Data Availability

Data are contained within the article.
